# Natural autoimmunity in oligoarticular juvenile idiopathic arthritis

**DOI:** 10.1186/s12969-023-00823-w

**Published:** 2023-05-03

**Authors:** Elena Tsitsami, Ioannis Sarrigeorgiou, Maria Tsinti, Erasmia C. Rouka, Sotirios G. Zarogiannis, Peggy Lymberi

**Affiliations:** 1 Pediatric Rheumatology Unit, First Department of Pediatrics, School of Medicine, University of Athens, Children’s Hospital “Aghia Sofia”, Thivon & Papadiamadopoulou, 11525 Athens, Greece; 2 Immunology Laboratory, Immunology Department, Hellenic Pasteur Institute, 127, Vasilissis Sofias Avenue, 11521 Athens, Greece; 3 Faculty of Nursing, School of Health Sciences, University of Thessaly, 41500 Geopolis, Larissa Greece; 4 Department of Physiology, Faculty of Medicine, School of Health Sciences, University of Thessaly, BIOPOLIS, 41500 Geopolis, Larissa Greece

**Keywords:** Oligo-JIA, Anterior Uveitis, Autoimmunity, Natural Antibodies, Antibody levels, ELISA

## Abstract

**Background:**

Oligoarticular juvenile idiopathic arthritis (oligo-JIA) is considered as an antigen-driven lymphocyte-mediated autoimmune disease. Natural antibodies (NAbs) are pre-immune antibodies produced in the absence of exogenous antigen stimulation, participating in both, innate and adaptive immunity. Considering their major immunoregulatory role in homeostasis and autoimmune pathogenesis, we designed this study to further elucidate their role in oligo-JIA pathogenesis.

**Methods:**

Seventy children with persistent oligo-JIA and 20 healthy matched controls were enrolled in the study. Serum IgM and IgA antibodies against human G-actin, human IgG F(ab΄)2 fragments and the hapten TriNitroPhenol (TNP) as well as the total concentration of serum IgM and IgA were measured by in-house enzyme-immunoassays. Kolmogorov–Smirnov normality test, Kruskal–Wallis H and Mann–Whitney tests were used to assess data distribution, and significant differences of non-parametric data between groups of the study. Backward regression analysis was used to analyze the effect of multiple factors (age, gender, disease activity, anti-nuclear antibody positivity, presence of uveitis) on continuous dependent variables (activities and activity/ concentration ratios of IgM and IgA NAbs).

**Results:**

The ratios of IgA anti-TNP, anti-actin and anti-F(ab΄)_2_ levels to total serum IgA concentration were found to be significantly increased in patients with oligo-JIA compared to healthy subjects. Significantly elevated levels of IgM anti-TNP antibodies were also found in children with inactive oligo-JIA compared to those of children with active disease and of healthy controls. In the presence of anterior uveitis, IgM anti-TNP levels were significantly higher than in patients without uveitis or in healthy controls. Backward regression analysis revealed that the disease activity and the presence of anterior uveitis independently affect IgM anti-TNP levels.

**Conclusuions:**

Our findings are in accordance with the hypothesis that NAbs contribute to the pathogenesis of autoimmune diseases and provide additional evidence that disturbances in natural autoimmunity may contribute to the as yet unclarified pathogenesis of oligo-JIA.

**Supplementary Information:**

The online version contains supplementary material available at 10.1186/s12969-023-00823-w.

## Background

Natural antibodies (NAbs) are defined as pre-immune antibodies produced spontaneously by distinct progenitors (B-1 cells) in the absence of exogenous antigen stimulation and providing immediate protection against infection while a specific and long-lasting adaptive response is mounting. Unique properties of NAbs setting them apart from antigen-specific antibodies are their broad cross-reactivity with a variety of epitopes expressed on both self and non-self antigens (autopolyreactivity), low affinity and germline structure (lacking non-templated nucleotides and little to no somatic hypermutation) [1]. IgM NAbs account for about 80% of all resting serum IgM and, therefore, are most extensively studied [2]. IgA NAbs represent about 50% of circulating IgA, while IgG and IgE NAbs have been also recognized. Beyond protection against various infections, IgM NAbs play fundamental roles in several diverse processes, including clearance of apoptotic debris, [3] regulation of B cell development [4, 5], selection of the B cell repertoire [6] and regulation of B cell responses [7]. It is believed that these functions of IgM NAbs help to maintain tissue homeostasis preventing, as a result, the development of autoimmune diseases [8, 9].

Oligoarticular juvenile idiopathic arthritis (oligo-JIA), the most common amongst the seven subtypes of JIA defined by the International League of Associations for Rheumatology (ILAR) [10], is classically thought of as an antigen-driven lymphocyte-mediated autoimmune disease [11, 12]. Despite that underlying immune pathways are yet to be elucidated, current evidence suggests that altered peripheral B cell homeostasis could be a factor significantly contributing to its pathogenesis [13, 14]. Changes in the frequency of autoreactive B cell subsets have been demonstrated that are accompanied by changes in the molecular events that control B cell tolerance. Indeed, CD5^+^ B cells, considered as the human counterparts of the murine NAbs producers B-1a cells [15], are expanded in the peripheral blood of patients with oligo-JIA [16]. Moreover, an expansion in CD24^hi^CD38^hi^ transitional B cells has been reported in oligo-JIA [17]. Like B-1 cells, a high proportion of these cells express polyreactive B cell receptors (BCRs), as they are still undergoing negative selection [18].

Bearing in mind the above, we designed this study to further elucidate the role of natural autoimmunity in the pathogenesis of oligo-JIA. Correlations between clinical parameters of the disease and NAb levels could also indicate their potential role as prognostic biomarkers in oligo-JIA. For this purpose, we examined levels of IgM and IgA NAbs against three selected antigens, namely TriNitroPhenol (TNP), G-actin and F(ab’)_2_ fragments, which share informative features associated with NAb recognition and their regulatory functions. More specifically, TNP was used for exogenous recognition, since it is a synthetic molecule to which individuals are not normally exposed [13]. Actin was used as an endogenous target, since it is a vital and abundant cytoskeleton component, largely preserved throughout evolution and a frequent target of NAbs [19, 20]. Last but not least, F(ab’)_2_ fragments were used as endogenous regulatory target, since Fab-Fab interactions participate in major immunoregulatory mechanisms [21], contributing to the termination of successful antibody responses and/or the control of autoreactive B cells [22, 23].

## Methods

### Patients enrolled in the study

Seventy (70) patients with persistent oligo-JIA diagnosed according to ILAR criteria, and twenty (20) healthy controls were enrolled in the study (Table 1). Patients with IgA immunodeficiency were excluded. Thirty-nine patients were in active disease and thirty-one with inactive. Active disease was clinically defined according to JADAS (Juvenile arthritis disease activity score assessment) 27 cut off scoring definition. Inactive disease state was defined as the presence of no joints with active arthritis, no systemic manifestations attributable to JIA, no active uveitis, normal levels of acute-phase reactants, a physician’s global assessment of disease activity indicating no disease activity, and duration of morning stiffness lesser or equal to 15 min [24, 25]. The presence or absence of disease activity was corroborated by Power Doppler ultrasonography performed by a pediatric radiologist. The Standardisation of Uveitis Nomenclature (SUN) criteria were used to define the anatomical location and time course of uveitis [26]. A valid analysis of the effect of treatment on NAb levels was not feasible, since the therapeutic modalities and schemes varied widely among patients. The study was approved by the Ethics Committee of the Children’s Hospital “Aghia Sofia”, and informed consent was obtained from the patient’s legal guardians.

**Table 1:** Patients and controls enrolled in the study

	**Patients**	**Controls**
N	70	20
Age (yrs)	8.1 ± 0.5	8.4 ± 0.8
Male/Female	12/58	7/13
Disease duration (yrs)	2.6 ± 3.4	
Active disease	39	
ANA ( +)	41	
Uveitis	18	

IgM and IgA antibodies against human skeletal muscle globular actin (G-actin), trinitrophenol-bovine serum albumin (TNP-BSA) conjugate and human IgG F(ab΄)_2_ fragments were measured in sera using in-house enzyme-linked immunosorbent assays (ELISA). In addition, total concentration for serum IgM and IgA was measured by ELISA. To evaluate the actual NAb-activity we took into consideration the total IgM/IgA concentration, in order to eliminate the possibility that higher NAb activity is due to increased IgM/IgA concentration. Therefore, in cases where significant differences were observed between groups with regard to the total IgM and/or IgA concentration, the ratio of NAb levels to the total IgM and IgA concentrations was used for comparisons instead of NAb levels.

### Antigens and antibodies

G-actin, TNP-BSA conjugate and F(ab΄)_2_ fragments derived from an IVIg preparation (Kiovig) were prepared as previously described [27, 28]. The purity and integrity of the above-mentioned antigens was evaluated by sodium dodecyl sulfate–polyacrylamide gel electrophoresis (SDS-PAGE). Antibodies used were unconjugated and alkaline phosphatase conjugated: goat antibodies recognizing human IgM (μ-chain specific) and human IgA (α-chain specific) (Sigma-Aldrich).

### Measurement of serum NAbs levels

IgM and IgA NAb activity in patients’ sera were evaluated by in-house ‘indirect’ ELISAs against G-actin, TNP-BSA and F(ab΄)_2_ fragments immobilized on high-binding, flat-bottomed 96-well MaxiSorp plates (Nunc, Denmark). Briefly, plates were 1) coated with antigens [10 μg/ml of human G-Actin, 10 μg/ml of TNP-BSA and 2 μg/ml of F(ab΄)_2_ fragments (in carbonate-bicarbonate buffer 0,1 M pH 9,6)], washed up and saturated with 1% BSA (Sigma-Aldrich) in PBS, 2) incubated with patients’ and healthy control sera [diluted (1/25 or 1/50, depending on the Ig isotype) in PBS containing 0.1% Tween and 1% BSA (PBS-T-BSA)] overnight at 4 °C, and washed, 3) incubated with alkaline phosphatase linked secondary antibodies (goat anti-human -μ, and -α chain) (0.5 μg/ml in PBS-T-BSA), for 2 h at 37 °C., and washed,, and 4) incubated with the enzyme’s soluble chromogenic substrate p-nitrophenyl phosphate disodium hexahydrate (pNPP) (Sigma-Aldrich) and optical density (OD) of colored product was measured at 405 nm (620 nm reference) with a TECAN spectrophotometer (TECAN Spark Control Magellan V2.2, Grödig/Salzburg, Austria). The enzyme reaction was stopped when the mean value of three selected positive controls reached OD equal to 1.00 (~ 1 h). As sera were tested in different microplates coated with the same antigen (at the same concentration and coating buffer), three standard control sera (treated as sample sera) were always added in each of the plates used to get normalization. All experiments were run in duplicate.

### Measurement of serum total IgM and IgA concentration

Human serum total IgM and IgA were measured by in-house ‘sandwich' ELISAs, following standard methodology. Briefly, goat anti-human IgM and IgA antibodies were used for coating (2.5 μg/ml in PBS) of microplates, and BSA (1% in PBS) for post-coating. Sera (diluted 1/4.000 for IgM and 1/15.000 for IgA), were incubated with coated plates, then AP-conjugated secondary anti-μ and anti-α antibodies were added. Dilution and wash buffers, as well as incubation time were as described above in NAbs-ELISA. An 8-point standard curve for IgM and IgA was included in each microplate using a standard control (N/T Protein Control SL/M, Siemens, Germany) (useful range: 500 to 3.9 ng/ml). OD values, reflecting IgM and IgA concentrations, were estimated according to the linear part of the standard curve.

### Statistical analysis

Statistical analysis was performed using the IBM SPSS v. 19.0 software. Graphical illustration was made in GraphPad Prism version 6.0.0. Data distribution was assessed with the Kolmogorov–Smirnov normality test. The Kruskal–Wallis H test was used to determine significant differences of non-parametric data between three groups followed by the Mann–Whitney test for two groups mean rank comparisons. Backward regression analysis was used to analyze the effect of multiple factors (age, gender, disease activity, anti-nuclear antibody (ANA) positivity, presence of uveitis) on continuous dependent variables (activities and activity/ concentration ratios of IgM and IgA NAbs). Statistical significance was set at the *p* < 0.05 level.

## Results

**Fig. 1:**
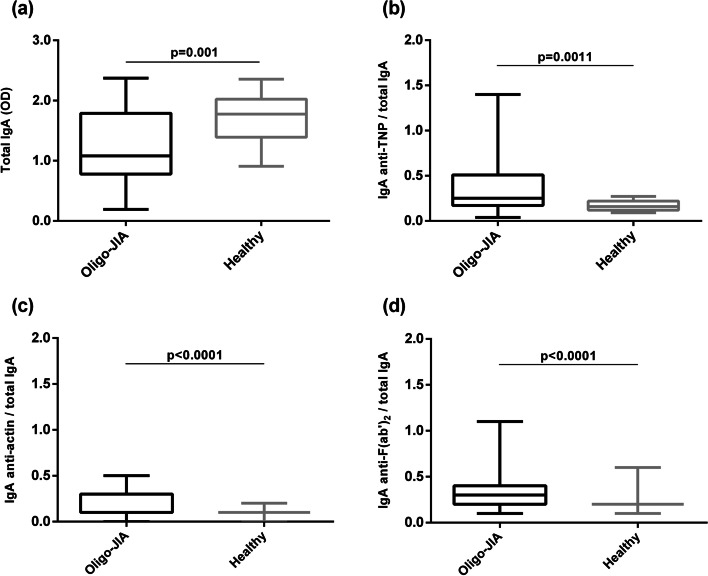
Measurements on IgA NAbs. Oligo-JIA patients (*n* = 70) and Healthy controls (*n* = 20) were measured for **a **total serum IgA concentration. Ratios of IgA NAb levels to total IgA concentration were estimated and analyzed for **b** anti-TNP, **c** anti-actin, and **d** anti-F(ab’)_2_ NAbs

IgM and IgA NAbs reactive to TNP, actin, and F(ab’)_2_ fragments were detected in the sera of oligo-JIA patients and matched healthy controls. All data analyzed in this study are included in supplementary information file 1 (SF1). Total IgA concentration in oligo-JIA patients was significantly lower compared to that of healthy controls (*p* = 0.001). The ratios of IgA anti-TNP, anti-actin and anti-F(ab΄)_2_ levels to total IgA concentration were significantly increased in patients with oligo-JIA compared to healthy subjects (*p* = 0.001, *p* < 0.001 and *p* < 0.001, respectively) (Fig. 1).

**Fig. 2:**
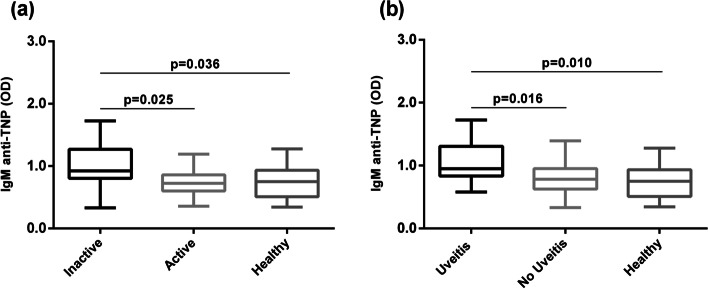
Measurements on IgM anti-TNP NAb levels. **a** Differences between oligo-JIA patients with active disease (*n* = 39) versus those with inactive disease (*n* = 31) and Healthy controls (*n* = 20). **b** Differences between oligo-JIA patients with uveitis (*n* = 18) against those without uveitis (*n* = 52) and Healthy controls (*n* = 20)

Differences in total serum IgM concentration were not detected among any group of patients or healthy controls. Females were observed to have significantly increased levels of IgM anti-TNP NAbs compared to males (*p* = 0.001). Significantly elevated levels of IgM anti-TNP NAbs were also found in children with inactive oligo-JIA compared to those of children with active disease (*p* = 0.025) and healthy controls (*p* = 0.036). In the presence of anterior uveitis, IgM anti-TNP levels was significantly higher than in patients without uveitis (*p* = 0.016) or in healthy controls (*p* = 0.010) (Fig. 2). Backward regression analysis showed significant associations between the IgM anti-TNP and anti-actin levels with anterior uveitis in the oligo-JIA group (*p* = 0.005/standardized coefficient 0.349 and *p* = 0.038/standardized coefficient 0.250, respectively). The same regression model identified an impact of both ANA and gender on the reactivity of the IgM isotype to the F(ab’)_2_ fragments (*p* = 0.016/standardized coefficient -0.298 and *p* = 0.028/standardized coefficient 0.272, respectively). No other associations were found among any other of the tested NAb levels or ratios and the parameters of gender and age, disease activity, ANA positivity and presence of anterior uveitis.

## Discussion

This is the first study on the role of NAbs in the context of persistent oligo-JIA. Significant changes in NAb concentration, repertoire and isotypes have been reported in many systemic and organ-specific autoimmune diseases while increasing evidence suggests that NAbs may interfere or even suppress autoimmune diseases and secondary events [29–32].

Because of the vast variety of antigens that are potential targets of NAbs, a combination of a minimum number of autoantibodies that reflects the state of the natural autoimmunity in humans is difficult to define [33]. Researchers have focused on different antigens, initially as a means to explore and classify the variety of the target antigens recognized by NAbs and then focused on some, due to their link with pathological conditions. We have selected three antigens, namely TNP, G-actin and F(ab’)_2_ fragments, as representative target antigens for the estimation of the natural autoimmunity status as: anti-TNP antibodies have been proposed as a surrogate measure for the estimation of serum polyreactivity, which is the key characteristic of NAbs; [34, 35] anti-actin antibodies have been found in sera taken from cord blood and healthy children but their implication in several diseases have been also reported in adult individuals; [18, 19, 36] studies on anti-F(ab’)_2_ autoantibodies in a variety of diseases, including autoimmune disorders, such as SLE, multiple sclerosis, nephritis, and others, support the experimental evidence regarding their immunoregulatory function [37–39].

In our study, significant differences in IgA concentrations were observed among oligo-JIA patients and healthy controls. Moreover, besides the differences in IgA concentration, patients with oligo-JIA presented higher levels of serum IgA NAbs against all the three antigens studied than those observed in age-matched healthy controls. Differences in the levels of IgA NAbs between oligo-JIA and controls observed in our study may reflect disturbances of the gut microbiome characterizing this subtype of JIA [40]. During the last decade, several studies uncover intestinal dysbiosis as a factor contributing to the pathogenesis of JIA subtypes, including oligo-JIA [41]. On the other side, systematically produced and locally secreted IgA NAbs seem to play a critical, despite incompletely understood [42], role in the gut microbiome homeostasis [43].

No differences in total IgM concentration were observed among oligo-JIA patients and healthy controls. Οn the contrary, significant differences in the levels of NAbs against TNP were found among oligo-JIA patients. Disease activity as well as anterior uveitis were proved as independent factors affecting serum levels of IgM anti-TNP NAbs. Indeed, IgM anti-TNP NAb levels in patients with inactive disease and/or anterior uveitis were higher than those of patients with active disease and/or without uveitis, the latter presenting levels similar to those of healthy controls.

Our finding that patients with active disease present with lower IgM NAb levels than those with inactive is in accordance with their contribution to the pathogenesis of autoimmune diseases [44, 45]. Several lines of evidence indicate the pivotal role of NAbs in the pathogenesis of autoimmune diseases [44, 45]. Mouse models of SLE have reduced levels of natural IgM [46, 47] with an increase in the B-1 cell subset and IgG autoantibodies [48, 49]. This reduction in natural IgM levels is associated with loss of the protective role of IgM NAbs, resulting in autoimmune diseases. In addition, in humans as well as in mice, deficiency in IgM increases an individual’s susceptibility for autoimmune diseases [50], while autoreactive IgM levels are greatly elevated in various autoimmune diseases, such as SLE, rheumatoid arthritis, and autoimmune liver diseases [3, 51, 52].

More interesting, however, is our result that the presence anterior uveitis affects independently the levels of circulating IgM NAbs. Similarly, previous studies have shown that in addition to ANA, serum anti-histone-3 antibodies and serum antibodies against ocular antigens are also associated with an increased risk of uveitis [53, 54]. This result signifies the possible involvement of natural autoimmunity in the as yet unclarified pathogenesis of this specific uveitis entity. Overall, the decrease observed in IgM NAb levels in active oligo-JIA patients and the increase of IgM NAb levels observed in uveitis patients may conceal informative features dependently on the systemic and/or the organ-specific form of autoimmunity that needs to be further investigated.

In our study, ANA positivity and gender independently affected IgM anti-F(ab’)_2_ levels, possibly indicating an imbalance in the idiotypic network resulting from the increase of ANA specificities as also previously found in patients with SLE [55, 56]. Additionally, an increase in anti-TNP IgM reactivity was observed in female subjects. These sex differences in IgM NAbs could be attributed to the fact that nutritional or other environmental exposures may vary by sex with potential long-term implications for immune-mediated disease as it has also been suggested by others [57].

Despite the limitations of the study in regard to the number of male controls and the effect of treatment, our results provide additional evidence that disturbances in natural autoimmunity may contribute to the as yet unclarified pathogenesis of oligo-JIA. It has been also shown that the three measured NAbs represent a panel satisfactorily describing the state of natural autoimmunity. Moreover, their independent association with disease activity and the presence of anterior uveitis indicates that it is worthy to be studied in appropriately designed epidemiological studies (including newly diagnosed treatment naïve patients, patients stratified according to therapeutic modalities used, etc.) in order to elucidate their possible use as prognostic biomarkers.

## Conclusions

- Disturbances in natural autoimmunity may contribute to the pathogenesis of oligo-JIA.
- Independent association of certain NAbs with disease activity and the presence of anterior uveitis indicates that they're worthy to be studied as possible prognostic biomarkers.

## Supplementary Information

**Additional file 1: Supplementary information file 1.** Demographic, clinical, and laboratory characteristics of all studyAQ participants.

## Data Availability

The datasets used and/or analyzed during the current study are available from the corresponding author on reasonable request.

## Abbreviations

JIA: juvenile idiopathic arthritis
oligo-JIA: oligoarticular idiopathic arthritis
ANA: anti-nuclear antibodies
NAbs: natural antibodies
TNP: trinitrophenol
OD: optical density
ELISA: enzyme-linked immunosorbent assay
