# Supplementary material for: Natural autoimmunity in oligoarticular juvenile idiopathic arthritis
Source: Pediatr Rheumatol Online J. 2023 May 3;21:44. doi: 10.1186/s12969-023-00823-w (PMC10155367; doi:10.1186/s12969-023-00823-w)
Supplement: Supplementary file 1 — Additional file 1: Supplementary information file 1. Demographic, clinical, and laboratory characteristics of all studyAQ participants. [file 12969_2023_823_MOESM1_ESM.pdf]

**Supplementary information file 1.** Demographic, clinical, and laboratory characteristics of all study participants											
	**Healthy Controls**<br>N=20<br>a	**Oligoarticular JIA**<br>N=70<br>b	**ANA (+)**<br>N=41<br>c	**ANA (-)**<br>N=29<br>d	**Uveitis**<br>N=18<br>e	**Non-Uveitis**<br>N=52<br>f	**Active disease**<br>N=39<br>g	**Inactive disease**<br>N=31<br>h	**Male**<br>N=19<br>i	**Female**<br>N=71<br>j	Mann-Whitney *p*- value
**Age, years, mean ± SEM ***	8,38±0,83	8,12±0,51	8,28±0,65	7,90±0,83	9,47±1,08	7,66±0,57	6,95±0,65	9,59±0,74	8,61±1,00	8,06±0,49	g vs h, *0.008*
**IgM concentration, mean ± SEM**	0,74±0,18	0,57±0,07	0,50±0,08	0,68±0,12	0,67±0,14	0,54±0,08	0,65±0,10	0,49±0,09	0,42±0,08	0,66±0,08	ns
**IgM TNP Activity, mean ± SEM**	0,75±0,05	0,86±0,03	0,86±0,05	0,87±0,05	1,03±0,07	0,80±0,03	0,79±0,04	0,95±0,06	0,67±0,06	0,88±0,03	a vs e, *0.010*<br>a vs h, *0.036*<br>e vs f, *0.016*<br>g vs h, *0.025*<br>i vs j, *0.001*
**IgM Actin Activity, mean ± SEM**	1,01±0,08	1,03±0,05	0,97±0,06	1,11±0,09	1,21±0,14	0,96±0,04	1,00±0,05	1,05±0,09	0,96±0,11	1,04±0,04	ns
**IgM F(ab´)$_2$ Activity, mean ± SEM**	1,22±0,10	1,32±0,05	1,24±0,06	1,44±0,09	1,45±0,11	1,27±0,06	1,32±0,07	1,32±0,07	1,02±0,06	1,37±0,05	i vs j, *0.004*
**IgA concentration, mean ± SEM**	1,8±0,16	1,21±0,78	1,06±0,09	1,41±0,13	1,33±0,13	1,17±0,10	1,30±0,12	1,13±0,10	1,53±0,19	1,32±0,08	a vs b, 0.001<br>a vs c, <0.0001<br>a vs e, 0.024<br>a vs f, 0.001<br>a vs g, 0.010<br>a vs h, 0.001<br>c vs d, 0.05
**Ratio (A/C) IgA TNP, mean ± SEM**	0,15±0,01	0,30±0,03	0,31±0,05	0,30±0,05	0,30±0,06	0,31±0,04	0,29±0,05	0,32±0,05	0,27±0,07	0,26±0,03	a vs b, 0.001<br>a vs c, 0.002<br>a vs d, 0.004<br>a vs e, 0.001<br>a vs f, 0.003<br>a vs g, 0.049<br>a vs h, <0.0001
**Ratio (A/C) IgA Actin, mean ± SEM**	0,09±0,01	0,19±0,01	0,18±0,02	0,20±0,03	0,17±0,03	0,20±0,02	0,17±0,02	0,21±0,02	0,16±0,04	0,17±0,01	a vs b, <0.0001<br>a vs c, 0.004<br>a vs d, <0.0001<br>a vs e, 0.020<br>a vs f, <0.0001<br>a vs g, 0.020<br>a vs h, <0.0001<br>g vs h, 0.042
**Ratio (A/C) IgA F(ab´)$_{2,}$ mean ± SEM**	0,22±0,02	0,45±0,04	0,46±0,06	0,43±0,06	0,40±0,06	0,47±0,06	0,44±0,07	0,47±0,05	0,33±0,07	0,40±0,04	a vs b, <0.0001<br>a vs c, <0.0001<br>a vs d, 0.002<br>a vs e, 0.001<br>a vs f, <0.0001<br>a vs g, 0.002<br>a vs h, <0.0001
